# Prevalence and avoidability of surgical adverse events in a teaching hospital in Brazil*

**DOI:** 10.1590/1518-8345.2939.3171

**Published:** 2019-10-07

**Authors:** Josemar Batista, Elaine Drehmer de Almeida Cruz, Francine Taporosky Alpendre, Denise Jorge Munhoz da Rocha, Marilise Borges Brandão, Eliane Cristina Sanches Maziero

**Affiliations:** 1 Universidade Federal do Paraná, Curitiba, PR, Brasil.; 2 Faculdades Santa Cruz, Curitiba, PR, Brasil.; 3 Bolsista da Coordenação de Aperfeiçoamento de Pessoal de Nível Superior (CAPES), Brasil.; 4 Universidade Federal do Paraná, Departamento de Enfermagem, Curitiba, PR, Brasil.; 5 Complexo Hospitalar de Clínicas da Universidade Federal do Paraná, Unidade de Centro Cirúrgico, Curitiba, PR, Brasil.; 6 Complexo Hospitalar de Clínicas da Universidade Federal do Paraná, Assessoria da Gestão da Qualidade, Curitiba, PR, Brasil.; 7 Governo do Estado do Paraná, Secretaria de Saúde do Estado do Paraná, Curitiba, PR, Brasil.

**Keywords:** Patient Safety, Medical Errors, Iatrogenic Disease, Surgical Procedures, Operative, Postoperative Complications, Surgical Wound Infection, Segurança do Paciente, Erros Médicos, Doença Iatrogênica, Procedimentos Cirúrgicos Operatórios, Complicações Pós-Operatórias, Infecção da Ferida Cirúrgica, Seguridad del Paciente, Errores Médicos, Enfermedad Iatrogénica, Procedimientos Quirúrgicos Operativos, Complicaciones Posoperatorias, Infección de la Herida Quirúrgica

## Abstract

**Objective:**

to estimate the prevalence and avoidability of surgical adverse events in a teaching hospital and to classify the events according to the type of incident and degree of damage.

**Method:**

cross-sectional retrospective study carried out in two phases. In phase I, nurses performed a retrospective review on a simple randomized sample of 192 records of adult patients using the Canadian Adverse Events Study form for case tracking. Phase II aimed at confirming the adverse event by an expert committee composed of physicians and nurses. Data were analyzed by univariate descriptive statistics.

**Results:**

the prevalence of surgical adverse events was 21.8%. In 52.4% of the cases, detection occurred on outpatient return. Of the 60 cases analyzed, 90% (n = 54) were preventable and more than two thirds resulted in mild to moderate damage. Surgical technical failures contributed in approximately 40% of the cases. There was a prevalence of the infection category associated with health care (50%, n = 30). Adverse events were mostly related to surgical site infection (30%, n = 18), suture dehiscence (16.7%, n = 10) and hematoma/seroma (15%, n = 9).

**Conclusion:**

the prevalence and avoidability of surgical adverse events are challenges faced by hospital management.

## Abstract (pt)

**Objetivo:**

estimar a prevalência e evitabilidade de eventos adversos cirúrgicos em hospital de ensino e classificar os eventos segundo o tipo de incidente e grau do dano.

**Método:**

estudo transversal e retrospectivo realizado em duas fases. Na fase I, enfermeiros realizaram revisão retrospectiva em amostra aleatória simples de 192 prontuários de pacientes adultos, utilizando o formulário do *Canadian Adverse Events Study* para rastreamento de casos. A fase II objetivou a confirmação do evento adverso por comitê de especialistas composto por médico e enfermeiros. Os dados foram analisados por estatística descritiva univariada.

**Resultados:**

a prevalência de eventos adversos cirúrgicos foi de 21,8%; em 52,4% dos casos, a detecção ocorreu no retorno ambulatorial. Dos 60 casos analisados, 90% (n=54) eram evitáveis e mais de dois terços resultaram em danos leves a moderados. Falhas técnicas cirúrgicas contribuíram em, aproximadamente, 40% dos casos. Houve prevalência da categoria infecção associada à atenção à saúde (50%;n=30). Destacaram-se os eventos adversos relacionados à infecção de sítio cirúrgico (30%;n=18), deiscência de sutura (16,7%;n=10) e hematoma/seroma (15%;n=9).

**Conclusão:**

a prevalência e evitabilidade dos eventos adversos cirúrgicos são desafios a serem enfrentados pela gestão hospitalar.

## Abstract (es)

**Objetivo:**

estimar la prevalencia y evitación de eventos adversos quirúrgicos en el hospital de enseñanza y clasificar los eventos según el tipo de incidente y grado del daño.

**Método:**

estudio transversal y retrospectivo realizado en dos fases. En la fase I, los enfermeros realizaron una revisión retrospectiva en una muestra aleatoria simple de 192 prontuarios de pacientes adultos, utilizando el formulario del *Canadian Adverse Events Study* para el seguimiento de casos. En la fase II, el objetivo tuvo la confirmación del evento adverso por comité de especialistas compuesto por médico y enfermeros. Los datos fueron analizados por estadística descriptiva univariana.

**Resultados:**

la prevalencia de eventos adversos quirúrgicos fue del 21,8%; en el 52,4% de los casos la detección ocurrió en el retorno al ambulatorio. De los 60 casos analizados, 90% (n=54) eran evitables y más de dos tercios resultaron en daños leves a moderados. Las fallas técnicas quirúrgicas contribuyeron en aproximadamente el 40% de los casos. La prevalencia de la categoría de infección asociada a la atención de la salud (50%, n=30). Se destacan los eventos adversos relacionados a la infección del sitio quirúrgico (30%, n=18), la dehiscencia de sutura quirúrgica (16,7%, n=10) e hematoma/seroma (15%, n=9).

**Conclusión:**

la prevalencia y evitación de los eventos adversos quirúrgicos son desafíos a ser enfrentados por la gestión hospitalaria.

## Introduction

The safety and quality of perioperative care are directly related to the development of techno-assistance models, posing challenges for health organizations due to the increasing technological evolution and incorporation of new clinical processes and surgical techniques. These advances contribute to the quality of the services provided to society. At the same time, they represent a health risk, which is exacerbated by structural failures of the system or by the deficiency in the management of work processes^1^, culminating in the occurrence of adverse events in patients submitted to surgical treatment.

The World Health Organization (WHO) defines adverse event (AE) as any incident that resulted in patient harm^2^ and presupposes that 230 million surgeries are performed annually in the world, with seven million AE and one million patients evolving to death^3^. There is a potential to avoid half of these cases in which surgery leads to damage^3^. This data fosters the need to adopt systematic practices for safe patient care in the perioperative period.

A systematic review identified a surgical AE rate of 14.4%^4^, while never events in North American surgical patients represented the occurrence of wrong-place surgery and retained surgical items of 1 AE/100,000 and 1 AE/10,000 procedures, respectively^5^. In Brazil, despite the lack of systematized data, a pioneering study conducted in three teaching hospitals in the Southeast region, with data from 1,103 admissions, in 2003, found an incidence of 7.6% of AE, among which 35.2% were attributed to surgical procedures^6^.

The AEs remain insufficiently investigated although they are a potential factor of morbidity and economic costs^7^, especially those related to surgical care. Studying the occurrence of surgical AEs constitutes a managerial tool that allows to recognize, implement, and evaluate improvement actions, and to organize and systematize the elements that make up the structure and the work process in health.

Thus, considering the demographic, epidemiological, and political-institutional transition at the national and regional levels, the importance of studies in this context as a strategy to encourage preventive actions is highlighted. These actions should be in consensus with the results of the 55th World Health Assembly, whose goals are to promote patient safety and quality of health care^3^.

In view of the foregoing, the present research was based on the guiding question: What is the prevalence, avoidability, and degree of damage of Surgical AEs in patients hospitalized in a teaching hospital in Brazil? Thus, the objective of this research was to estimate the prevalence and avoidability of surgical AEs in a teaching hospital in Brazil and to classify them according to the type of incident and degree of damage.

## Method

This is a cross-sectional and retrospective study developed in a high-complexity public teaching hospital located in southern Brazil. The hospital has more than 600 beds funded by the Unified Health System and performs, on average, 840 surgeries/month. In 2010, the use of the Surgical Safety Checklist proposed by the WHO “Safe Surgery Saves Lives” initiative was implemented. During the second half of 2014, another checklist was implemented, applied to the surgical hospitalization units and complementary to the previous one, containing 97 safety indicators organized into six categories: identification, preoperative period, immediate postoperative period, mediate postoperative period, other surgical complications, and hospital discharge^8^.

The sample was composed of medical records of patients submitted to surgical procedures hospitalized in the units of orthopedics, general surgery, digestive system surgery, neurological surgery, plastic surgery, and hepatic transplantation, from June 2014 to May 2015. The first procedure, which corresponded to the index hospitalization, of patients aged ≥18 years and with a minimum hospital stay of 24 hours was analyzed. Following the criteria adopted by previous studies^9-10^, medical records of psychiatric patients were excluded.

A total of 2,593 medical records were eligible for the study. The parameters used to define the sample size were based on the incidence of surgical complications of 16%^3^, sample error of 5%, and level of significance of 5% whose calculation resulted in 192 medical records. The random selection was performed based on the list of surgeries issued by the institution’s computer service. The medical records that were ineligible or unavailable in the filing service were replaced by the immediately subsequent medical records of the general list of surgeries.

The identification of the occurrence of the AE and its avoidability was employed through a retrospective review of medical records based on a protocol from the Canadian Adverse Events Study (CAES), which advocates the identification and analysis of AEs in two phases^9^. Phase I refers to the screening of potential adverse events (pAE) guided by explicit criteria, which was performed through double review of medical records by two nurses with experience in the surgical area, using the screening form, translated and adapted for the Brazilian context^10^.

This form includes 17 criteria for tracking pAE related to surgical and anesthetic procedures, drug use, diagnosis, non-drug care and treatment. Considering the population of this research, the trackers related to miscarriage, labor, and delivery were excluded, using 16 trackers originating from the original list^9-10^. For the identification of pAE related to surgical site infection (SSI) occurring after hospital discharge, the records contained in the records of outpatient consultations were used, as well as the criteria recommended by the Centers for Disease Control and Prevention, which defined SSI as the one that occurs within 30 days after the surgical procedure and/or 90 days after implant insertion^11^.

Upon detecting the presence of at least one screening criterion, regardless of which tracer, a semi-structured script was completed to characterize the demographic, clinical, surgical, and anesthetic profile. Subsequently, the pAE investigation form was completed and the record was included for review in phase II. This refers to the confirmation or otherwise of the AE through implicit structured review, which was performed by a physician and two nurses with more than 20 years of experience in the area of quality management and patient safety.

This group composed the committee of experts with the objective of judging the occurrence or not of the AE, in consensus guided by the definition of the term by WHO^2^ and with the use of two scales. The first scale was to judge whether the AE was caused by patient care and the second scale was to assess the degree of avoidability. The scales have six points, and experts considered an event as an AE and with potential of avoidability when the score reached ≥4 points^6,9^. Surgical events were classified as highly preventable, potentially preventable, potentially non-preventable and highly non-preventable^6,9^.

The AE were classified according to degree of physical damage as mild, moderate, severe, and fatal, and according to the International Classification for Patient Safety as class 1 (type of incident), consisting of the following categories: clinical administration; clinical process/procedure; documentation; healthcare-related infection (HCRI); intravenous medication/fluids; blood/blood products; nutrition; oxygen/gases/vapors; medical devices/equipment; behavior; accidents with the patient; infrastructure/location/facilities; and organizational resources/management^2^.

The measures used were prevalence of surgical AEs among inpatients [(number of patients with at least one surgical AEs/total number of patients) x 100] and the proportion of preventable surgical AEs [number of preventable surgical AEs/total number of surgical AEs] x 100]. The data collected was transferred to a Microsoft Office Excel 2016® spreadsheet with double typing for validation and checking for consistency. Univariate descriptive statistical analysis was performed using the IBM SPSS 20 software (Statistical Package for the Social Sciences).

This research belongs to the thematic project: “Evaluation of safety culture and occurrence of surgical adverse events in Brazilian hospitals”. It met the ethical precepts of research involving human beings and was approved by the Institutional Research Ethics Committee under number 1.990.760.

## Results

**Figure 1:**
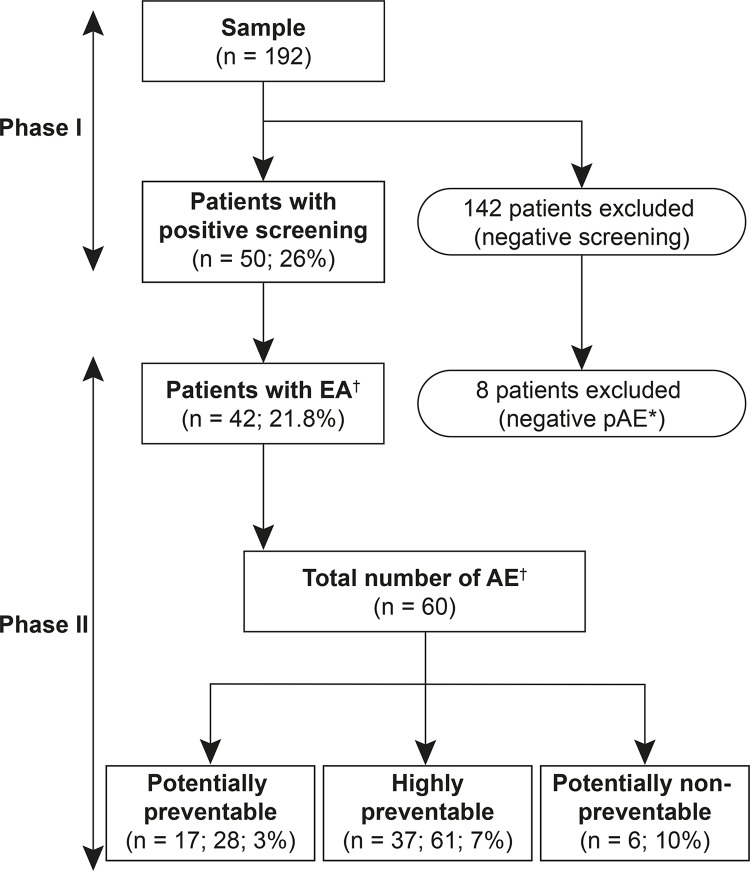
= Flowchart for the selection of analyzed records and estimation of the prevalence and avoidability of surgical adverse events. Curitiba, PR, Brazil, 2017

The frequency of records with positive screening for pAE, prevalence rate, and avoidability of cases are presented in Figure 1. Out of the 42 surgical patients affected by AEs, 26.2% (n = 11) had more than one occurrence, totaling 60 Surgical AEs, of which 90% (n = 54) were classified as preventable.

*pAE = potential adverse event; ^†^AE = adverse event

Among the surgical patients with AE, the mean age was 44.5 years (standard deviation - SD ± 15.5) and the mean hospitalization time was 11.9 days (SD ± 21.1), ranging from one to 102 days. The hospitalizations were related to elective surgical procedures and, among the clinical conditions, patients had mild systemic disease. The most frequent comorbidities/risk factors were severe hypertension (33.3%, n = 14), smoking (23.8%, n = 10), diabetes *mellitus* (11.9%, n = 5), and obesity (9.5%, n = 4). The other demographic, surgical, and anesthetic characteristics of patients with surgical AEs are presented in Table 1.

**Table 1:** Demographic, surgical, and anesthetic characteristics of patients with surgical adverse events. Curitiba, PR, Brazil, 2017

Variable	n	%
Sex		
Male	24	57.1
Female	18	42.9
Age range		
< 60 years	33	78.6
≥ 60 years	9	21.4
Preoperative time of hospitalization		
< 24 hours	37	88.1
≥ 24 hours	5	11.9
Hospitalization time		
1 to 2 days	16	38.1
3 to 5 days	9	21.4
≥ 6 days	17	40.5
Surgical classification		
Elective	36	85.7
Emergency	6	14.3
Degree of contamination		
Clean	16	38.1
Potentially contaminated	11	26.2
Contaminated	12	28.6
Infected	3	7.1
Duration of surgery		
< 120 minutes	22	52.4
≥ 120 minutes	20	47.6
Surgical risk ASA*		
ASA^*^ I^†^	7	16.7
ASA* II^‡^	26	61.9
ASA* III^§^	9	21.4
Type of Anesthesia		
Spinal anesthesia	16	38.0
General	15	35.7
Combined^||^	7	16.7
Epidural	2	4.8
Local	2	4.8

*ASA = American Society of Anesthesiology; ^†^I = Healthy patient; ^‡^II = Patient with mild systemic disease; ^§^III = Patient with severe systemic disease without risk of death; ^||^Combination of two or more types of anesthesia

The surgical procedures hernioplasty, knee/hip arthroplasty, appendectomy, and cholecystectomy were the ones that most frequently evolved with AE (47.6%, n = 20); videolaparoscopic surgeries accounted for 14.3% of the cases (n = 6).

Surgical AEs were concentrated in the category of healthcare-associated infection associated, with 50% of the cases (n = 30), followed by clinical procedure/process (38.4%, n = 23), accidents with the patient (8.3%, n = 5), and medical device/equipment, with 3.3% (n = 2), as presented in Table 2. Regarding the degree of physical damage to patients who were affected by AE, 90% (n = 54) were classified as mild and/or moderate. In the records analyzed, no AE with death outcome was detected.

**Table 2:** Distribution of surgical adverse events according to type of incident, degree of damage, and potential of avoidability. Curitiba, PR, Brazil, 2017

Variable			Degree of damage			Potential of avoidability		
			
			Mild	Moderate	Severe	HP*	PP^†^	PNP^‡^
			
Adverse event	n	%	n (%)	n (%)	n (%)	n (%)	n (%)	n (%)
Surgical Site Infection	18	30.0	7 (38.9)	10 (55.5)	1 (5.6)	13 (72.2)	5 (27.8)	0 (0.0)
Dehiscence	10	16.7	7 (70.0)	3 (30.0)	0 (0.0)	8 (80.0)	1 (10.0)	1 (10.0)
Hematoma/Seroma	9	15.0	5 (55.6)	3 (33.3)	1 (11.1)	7 (77.8)	2 (22.2)	0 (0.0)
Urinary retention	5	8.4	5 (100)	0 (0.0)	0 (0.0)	0 (0.0)	0 (0.0)	5 (100)
Deep vein thrombosis	3	5.0	0 (0.0)	3 (100)	0 (0.0)	0 (0.0)	3 (100)	0 (0.0)
Perforation /Laceration	3	5.0	0 (0.0)	2 (66.6)	1 (33.3)	2 (66.6)	1 (33.3)	0 (0.0)
Skin/mucosal injury	2	3.3	1 (50.0)	1 (50.0)	0 (0.0)	2 (100)	0 (0.0)	0 (0.0)
Sepsis/Septic shock	2	3.3	1 (50.0)	1 (50.0)	0 (0.0)	1 (50.0)	1 (50.0)	0 (0.0)
Fistula	2	3.3	0 (0.0)	2 (100)	0 (0.0)	0 (0.0)	2 (100)	0 (0.0)
Hemorrhage	2	3.3	0 (0.0)	1 (50.0)	1 (50.0)	1 (50.0)	1 (50.0)	0 (0.0)
Falls	2	3.3	1 (50.0)	0 (0.0)	1 (50.0)	2 (100)	0 (0.0)	0 (0.0)
Hypoxia	1	1.7	0 (0.0)	0 (0.0)	1 (100)	1 (100)	0 (0.0)	0 (0.0)
Hoarseness	1	1.7	1 (100)	0 (0.0)	0 (0.0)	0 (0.0)	1 (100)	0 (0.0)
Total	60	100	28 (46.7)	26 (43.3)	6 (10.0)	37 (61.7)	17 (28.3)	6 (10.0)

*HP = Highly preventable; ^†^PP = Potentially preventable; ^‡^PNP = Potentially non-preventable

Outpatient return records contributed to 52.4% (n = 22) of the cases, and of the total of these patients, two (4.8%) were readmitted as a result of the AE.

## Discussion

Despite the limitations inherent to the retrospective review of medical records, it allowed identifying the prevalence of potentially preventable surgical AEs in a single hospital setting. The results presented here raise reflection on the possible magnitude of the problem in Brazil, especially in less economically privileged regions, considering geographic and regional inequalities in the provision of surgical care, as well as the availability of qualified professionals^12^.

The prevalence of 21.8% of surgical AEs found in the present study was higher than that registered in research conducted in Sweden (15.4%)^13^, in a university hospital in Japan (15.1%)^14^, and falls short of a study carried out in Spain with patients submitted to general surgery (36.8%)^15^. In Brazil, in a study carried out in three hospitals in the Southeast region, the incidence of surgical AEs was 3.5%^16^, while in Europe, in a study in 30 public acute care hospitals care hospitals, the incidence was 13.1%^17^.

The literature points out that the performance of the reviewers may be one of the factors related to underestimation of cases^18^. However, the frequency of pAE identified in the present study was similar to the performance of reviewers whose primary revision reached 21.6% of positive screening^18^ and fell short of Swedish reviewers who found 34.3% of positive records with pAE for inclusion in phase II^19^. One of the factors for the occurrence of underestimation of trackers in this research was incomplete, illegible, and erased annotations/records, which was possibly aggravated by the institutional use of physical records.

The avoidability of surgical AEs was higher than the values reported in several studies, ranging from 5.2% to 70.8%^4,13,15-17,20^, which raises the need to evaluate, at the same time, indicators of surgical care. It also may encourage managers, surgeons, and nursing staff, among others, to reassess the care process and to propose actions for continuous improvement.

Apart from the geographic differences, the methodological designs used in different researches, and the quality of the services provided in different regions and countries of the world, there is evidence of the vulnerability of patients to the occurrence of one or more surgical AEs. These AEs are mostly preventable, as pointed out by previous studies^13,16^ and reinforced by the present study, which identified that 26.2% of the patients (n = 11) suffered more than one AE during the index hospitalization.

These findings reveal that errors and failures in the surgical care process can cause several incidents in the same individual, resulting in physical damage. A systematic review showed that mild and moderate damage corresponded to 86.7% of cases^4^. These data are consistent with the results presented here, in which more than two-thirds of the events resulted in mild to moderate disability. This reinforces the principle of the second global challenge in patient safety (Safe Surgery Saves Lives), as well as the use of the Surgical Safety Checklist by the health services, which contributes to the reduction of AE in the surgical environment^3^.

The studied institution implanted the surgical checklist and also developed a checklist to be applied in the hospitalization units by the nursing team in the preoperative and postoperative periods^8^. However, the results emphasize surgical AEs associated to technical failures during the execution of the surgical procedure (hematoma/seroma, dehiscence, perforation/laceration, wall necrosis, hemorrhage and gas embolism), contributing to approximately 40% of the cases. This data diverges from another study that reported AE related to errors in the hospitalization management in a higher proportion than to the surgical technique^4^.

Thus, the results point mainly to the need for review/improvement of the operative technique and are consistent with the findings of another Brazilian study, which pointed out 27% (n = 7) of technical failures in a surgical center^21^, in the same way as in a medical center in China, in which a study showed 61.6% (n = 16) of AEs related to technical and/or surveillance failures^22^.

Therefore, because it is a teaching hospital, with professionals improving their clinical and surgical skills, constant training and supervision is essential with a view to promoting the quality of surgical care and correcting nonconformities. In this study, approximately 10% of the cases were found to be severe AEs, which was higher than the percentage of an American study that analyzed 676 surgical surgeries and found a prevalence of 6.36% (n = 43) of severe events^20^. In a Brazilian study, 21.9% (n = 9) of the cases presented permanent damage, of which 17.1% (n = 7) evolved to death^16^.

Data from other investigations have indicated that AEs are more frequent among elderly patients^13,19^, differently from what occurred in this study, whose highest prevalence was among patients in the age group between 18 and 59 years of age. This fact can be justified, firstly, by the service profile of surgical units, predominantly composed of young adults, low prevalence of severe systemic disease, and absence of risk factors/comorbidities. Added to this, there is the predominance of elective surgeries, in which occurs better surgical preparation, as well as the lower risk of incidents related to the younger population.

Surgical AEs were related to HCRI in 50% (n = 30), and SSI represented almost one-third of these. These events are considered the most common among surgical patients, despite the various evidence-based strategies that can be implemented to reduce them^23^, as well as the use of the Surgical Safety Checklist, whose adaptation to the institutional context was performed for SSI prevention^24^. Thus, basic measures and recognized as scientific evidence are recommended by international institutions and corroborated in Brazil and should be part not only of surgical protocols, but also of audit for the quality of care.

Another factor to consider in SSI prevention focuses on the safety culture of the unit, evidenced in a cross-sectional study conducted in seven American hospitals that associated culture scores with the reduction of SSI rates in colon surgeries^25^. In view of the high prevalence of AEs related to SSI, there should be evaluation of the indicators of surgical assistance that increase the risk of its occurrence^11,23^ and raises reflection on the safety culture and financial waste in the Brazilian health system, considering that, most SSIs were considered as strongly or potentially avoidable.

The surgical suture dehiscence had low prevalence in an American study, which analyzed 676 surgical surgeries and found two cases^20^; however, it represented 3.67% (n = 8) of the AEs in a Brazilian study^26^. These data contrast with the results of this research, in which this event was the second most frequent, with a prevalence of 16.7% (n = 10), and indicates the need to evaluate, in addition to professional technical ability, the possibility of technical problems with the material used to perform the procedure.

The third most prevalent AE was associated with hematomas/seromas and represented, in a Spanish study, 8.9% (n = 16) of patients submitted to general surgery^15^. This AE, if not treated properly, can cause physical discomfort and increase the risk of infection^27^, besides compromising the cicatrization process and predisposing the patients to surgical wound suture dehiscence. To avoid this AE, a set of actions related to surgical technique and postoperative care should be adopted.

Deep venous thrombosis occurred in three patients (5%), a percentage higher to that identified in a study conducted in Japan (1%, n = 3)^14^. There are several measures to avoid this AE and they are widely recognized, ranging from the identification of high-risk patients, drug prophylaxis, early ambulation, and the use of compressive stockings. Practitioners should establish and strictly follow a prophylaxis protocol for thromboembolism, since the literature indicates that the inability to implement or follow a protocol is a contributory factor for the occurrence of AE^16,18^, becoming a limiting human factor for patient safety and prevention of surgical AEs.

Regarding organ perforation/laceration, a prevalence of 5% (n = 3) was observed, and it was mostly associated with central venous puncture. This can be potentially avoidable with the use of ultrasonography during the procedure. In a hospital in Texas, USA, lacerations represented 7.1% (n = 48), of which 6.5% were preventable and almost half of the cases prolonged the hospitalization time of the patient^20^.

Skin and mucosal lesions, whether due to surgical positioning, bed rest, allergic reaction to medical-hospital patches, or clinical procedures can be prevented when properly approached by the health team. The analysis of 507 AEs in surgical units from 63 hospitals in Sweden found that these lesions affected 31 patients (6.1%) and 94% were considered preventable^13^. In this sense, using scales for risk stratification resulting from surgical positioning^28^ can be a feasible strategy to minimize the occurrence of this AE.

Sepsis/septic shock accounted for 3.3% of AEs in this study, falling short of that found in the Swedish study, with 13.2% (n = 30)^13^. Because it is considered a severe AE and poses a risk to the patient’s life, professionals’ qualification for the identification of predictive signs and early diagnosis is highlighted as a strategy to avoid it. For this purpose, studies are necessary to determine the causes with a view to prevention of this AE.

Falls and hemorrhage represented a prevalence of 3.3% (n = 2), each. In a Brazilian study, hemorrhages accounted for 12.2% (n=5) of AEs^16^, while in a study analyzing acute care hospitals in the United States, with a sample of 676 patients, the prevalence was 35.6% (n=241)^20^. The low prevalence of this AE in the present study may be related to the inaccuracy of the records. It is believed that the training for excellence in written communication and the use of a standardized instrument for accurate measurement may contribute to the identification of cases and serve as a basis for therapeutic conducts.

Falls had a prevalence of 2.7% and 2.4% of the surgical patients attended in a surgical unit in Brazil and Sweden, respectively^13,26^. In the hospital of the present research, there is a protocol for the prevention of falls, but the constant need for improvement is highlighted, especially because of the risk posed to the patient in the postoperative period.

The AEs considered potentially non-preventable were mainly related to urinary retention after the use of opioids or postoperative analgesia by epidural catheter. A study conducted in eight acute care hospitals in Texas, USA, found that 92.5% (n = 37) of 40 surgical surgeries related to urinary retention were classified as non-preventable^20^.

Urinary retention predisposes to risks of urinary tract infection, since it often requires additional therapy, such as bladder catheterization, in addition to the risk of prolonged urine retention that predisposes to microbial proliferation. However, aggressive pain management is crucial because the consequences of ineffective treatment of acute pain are often greater than the risk of adverse side effects from the use of analgesics^29^. Improving the preoperative evaluation by the multidisciplinary team and identifying the intrinsic risk factors may contribute to better preoperative planning and reduction of cases of urinary retention.

It was also noteworthy in this research the high detection of AEs through outpatient return records, with two readmissions. It has been proven that AEs increase hospitalization time, with consequent increase in hospital costs^7,16^, as well as outpatient return and early emergency care interventions. This finding reiterates the need to develop strategies for surgical surveillance after discharge, whose objective is to identify events beyond the hospital’s internal environments, which may include an active notification system. These data may support preventive measures, improve the diagnosis of patient safety, as well as the progressive development of organizational safety culture, becoming elements to be managed by the units studied and the hospital organization.

The present study has some limitations. One of them is that the results come from a retrospective review of records of a single hospital environment, which prevents the generalization of the results. The records had not been fully completed by the medical and nursing staff, which may have interfered in the detection of AEs. In some cases, the death outcome occurred at home and/or other hospital institution, making it impossible to investigate the screening criteria. Another limiting factor was the lack of uniformity in research and classification methods for the detection, analysis, and confirmation of AEs, which make it difficult to compare these results between different healthcare contexts.

Despite these limitations, this study has strengths. The first one focuses on the use of a standardized international methodology for the search and confirmation of AEs and the incipience of studies to estimate the prevalence of AEs in a specific population of surgical patients of a Brazilian teaching hospital. In addition, the study is a pioneer in the country in investigating the surgical AEs occurred during hospitalization and after discharge with outpatient return. Another strength is related to the phase of confirmation and analysis of the AEs, which was achieved through consensus of a panel of experts, allowing to avoid the undue discard of tracked records and reduce the subjectivity in the judgment of the cases.

## Conclusion

The findings showed that approximately half of the surgical AEs were identified on outpatient return, caused mild to moderate damage, and were mostly classified as preventable. The most prevalent surgical AEs were HCRI, with emphasis on SSI, which represented almost one-third of all cases. The prevalence and avoidability of surgical AEs in this research are challenges to be faced by hospital management in the surgical context.

This study is expected to stimulate the investigation of the prevalence of surgical AEs in different care contexts and may contribute to the implementation of safe health practices, with a view to promoting the quality of care, according to international recommendations and national guidelines.
